# *C. elegans* kinesin 1 regulates meiotic spindle size

**DOI:** 10.17912/micropub.biology.001820

**Published:** 2025-09-13

**Authors:** Rebecca Do, Alma Aquino, Francis McNally

**Affiliations:** 1 Molecular and Cellular Biology, University of California, Davis, Davis, California, United States

## Abstract

During *C. elegans* female meiosis, kinesin 1 transports yolk granules inward which appears to indirectly drive outward movement of the meiotic spindle to the cortex. Here we examined the size of the centrally located metaphase I meiotic spindle in a germline null allele of kinesin 1 heavy chain. Centrally located meiotic spindles were both longer and wider in kinesin germline null embryos compared with cortical meiotic spindles in controls. Spindle size might be minimized by cortically localized signaling machinery. Alternatively, kinesin 1 which localizes on the spindle during metaphase, may act directly on microtubules to minimize spindle size.

**Figure 1. Deletion of unc-116 results in an enlarged metaphase I spindle:**
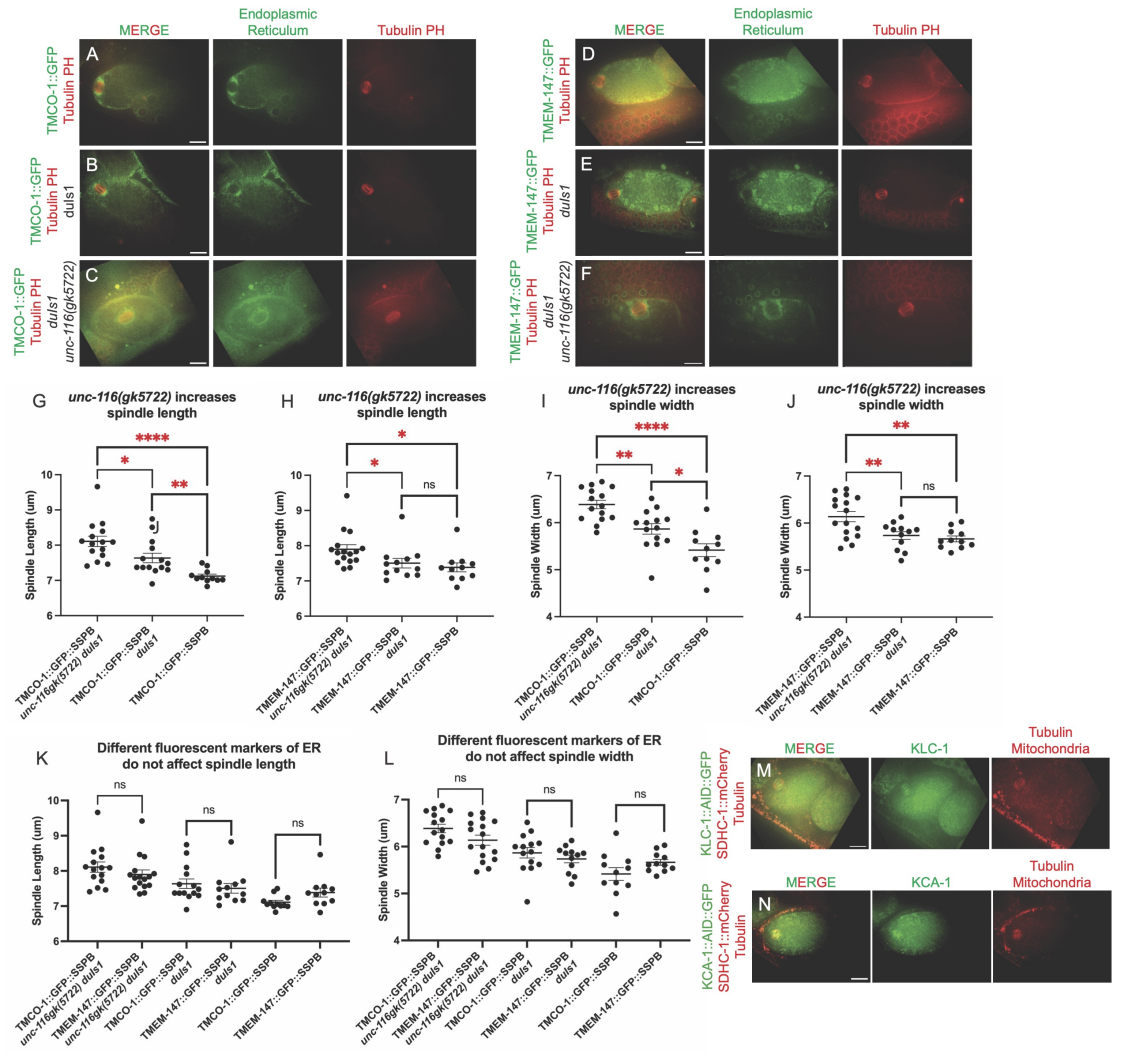
(A-F) Single plane images from time-lapse sequences of metaphase I embryos filmed in utero. (G-L) Comparison of metaphase I spindle lengths and widths. (M) Wildtype meiotic embryo showing KLC-1 ::AID::GFP (green) localized in the spindle midzone during metaphase I. (N) Wildtype meiotic embryo showing KCA-1 ::AID::GFP (green) localized in the spindle midzone during metaphase I. Statistics: Student's t test (*p<0.1; **p<0.01; ****p<0.0001). All scale bars = 10 μm.

## Description

In cultured mammalian cells where microtubule plus ends extend outward from a centrosome, kinesin 1 participates in the outward movement of the endoplasmic reticulum, ER (Wozniak et al., 2009). During ovulation of *C. elegans* oocytes, kinesin 1 transports yolk granules on microtubules whose plus ends extend inward from the plasma membrane (McNally et al., 2010). Premature activation of kinesin 1 causes early inward packing of yolk granules into a discrete ball in diakinesis oocytes and the ER is excluded from this ball, suggesting that inward transport of yolk granules can generate an outward force on the sheet-like ER that envelopes the meiotic spindle and that the ER is not directly bound by kinesin 1. This interpretation is supported by the finding that the kinesin 1 subunits, KLC-1 and KCA-1 , are localized on the surface of yolk granules during ovulation, then transfer to microtubules after ovulation (Aquino et al., 2025).

To test whether cortical meiotic spindle positioning might affect spindle morphology, we monitored spindle size in control vs the centered spindle in a germline null allele of *unc-116* , kinesin 1 heavy chain [ *unc-116 ( gk5722 ); duIs1* ]. *unc-116 ( gk5722 )* is a larval lethal deletion allele and its larval arrest is overcome by *duIs1* , an integrated multi-copy array of *unc-116 ::GFP* that is silenced in the germline. To determine whether kinesin 1 affects spindle geometry, we monitored the length and width of metaphase I spindles. Metaphase I spindles maintain a constant length for approximately 7 min after ovulation before shortening (Yang et al., 2005) so all measurements were taken from time-lapse sequences shortly after ovulation ( Fig. 1A- F). Spindles were significantly longer than controls ( Fig. 1G, 1H) and significantly wider than controls ( Fig. 1I, 1J) in *unc-116 ( gk5722 ); duIs1* metaphase I embryos. In some cases, there was an increase in spindle length ( Fig. 1G ) and width ( Fig. 1I ) just from the *duIs1* transgene which is not expressed in the germline. This might indicate that the integrated array is causing partial cosuppression (Dernburg et al., 2000). Two different ER markers were used for this study, F22B5.10 is a homolog of human TMCO1 and ZK418.5 is a homolog of human TMEM147. Spindle length and width did not depend on which ER marker was expressed ( Fig. 1K, 1L).

Two hypotheses could explain the increased spindle size in the absence of kinesin 1. First, signaling machinery at the cortex might activate microtubule disassembly factors in cortical but not centered spindles. Second, the kinesin 1 subunits, KLC-1 ( Fig. 1M ) and KCA-1 ( Fig. 1N ), transferred from yolk granules to spindle microtubules during ovulation (10/10 time lapse sequences each) indicating that kinesin 1 might directly affect spindle microtubules possibly through microtubule-microtubule sliding (Lu et al., 2015).

## Methods

For time-lapse imaging, anesthetized worms were mounted between an agarose pad and coverslip as described in (Danlasky et al., 2020) and subjected to single plane time-lapse imaging on a Yokogawa CSU-10 spinning disk confocal microscope equipped with an Olympus 100X 1.3 PlanApo objective and a Hammamatsu Orca Quest qCMOS detector. Exposures were captured every 5 seconds. Metaphase spindle length and width measurements were collected on spindles before the initiation of shortening.

## Reagents

**Table:** 

Strain number	genotype	Available from
FM969	*duSi29* [pFM1994; F22B5.10 ::GFP(GLO)::SSPB(nanoGLO)]II; *unc-116* ( *gk5722* [loxP + *myo-2* p::GFP:: *unc-54* 3' UTR + *rps-27* p::neoR:: *unc-54* 3' UTR + loxP])III; *ItIs44* [ *pie-1* p-mCh::PH(PLC1delta1) + *unc-119* (+)]V; *duIs1* [ *unc-116* ::GFP integrated array]; *wjIs76* [Cn_unc-119(+); *pie-1* p::mKate2:: *tba-2* ]	fjmcnally@ucdavis.edu
FM1221	*duSi29* [pFM1994; F22B5.10 ::GFP(GLO)::SSPB(nanoGLO)]II; [ *pie-1* p-mCh::PH(PLC1delta1) + *unc-119* (+)]V; *wjIs76* [ *Cn_unc-119* (+); *pie-1* p::mKate2:: *tba-2* ]	fjmcnally@ucdavis.edu
FM1292	*duSi29* {pFM1994[TMCO-1::GFP(GLO)::SSPB(nanoGLO)]II}; *duIs1* [ *unc-116* ::GFP integrated array]; *ItIs44* [ *pie-1* p-mCh::PH(PLC1delta1) + *unc-119* (+)]V; *wjIs76* [ *Cn_unc-119* (+); *pie-1* p::mKate2:: *tba-2* ]	fjmcnally@ucdavis.edu
FM1134	*duSi31* [pFM1968; ZK418.5 ::GFP::SSPB(nanoGLO) II; *unc-116* ( *gk5722* [loxP + *myo-2* p::GFP:: *unc-54* 3' UTR + *rps-27* p::neoR:: *unc-54* 3' UTR + loxP])III; *ItIs44 [ pie-1* p-mCh::PH(PLC1delta1) + *unc-119* (+)]V; *duIs1* [ *unc-116* ::GFP integrated array]; *wjIs76* [ *Cn_unc-119* (+); *pie-1* p::mKate2:: *tba-2* ]	fjmcnally@ucdavis.edu
FM1267	*duSi31* [pFM1968; ZK418.5 ::GFP::SSPB(nanoGLO) II; *ItIs44* pAA173; [ *pie-1* p-mCh::PH(PLC1delta1) + *unc-119* (+)]V; *wjIs76* [ *Cn_unc-119* (+); *pie-1* p::mKate2:: *tba-2* ];	fjmcnally@ucdavis.edu
FM1293	*duSi31* [pFM1968; ZK418.5 ::GFP::SSPB(nanoGLO) II; *ItIs44* [ *pie-1* p-mCh::PH(PLC1delta1) + *unc-119* (+)]V; *duIs1* [ *unc-116* ::GFP integrated array]; *wjIs76* [ *Cn_unc-119* (+); *pie-1* p::mKate2:: *tba-2* ]	fjmcnally@ucdavis.edu
